# Type B Uncertainty Analysis of Gravity-Based Determinations of Triaxial-Accelerometer Properties by Simulation of Measurement Errors

**DOI:** 10.6028/jres.126.038

**Published:** 2022-01-22

**Authors:** Jon Geist, Michael Gaitan

**Affiliations:** 1 National Institute of Standards and Technology, Gaithersburg, MD 20899 USA

**Keywords:** acceleration, accelerometer, gravity, inertial, measurement, rotation, triaxial

## Abstract

We simulated the effects of gimbal-alignment errors and rotational step-size errors on measurements of the sensitivity matrix and intrinsic properties of a triaxial accelerometer. We restricted the study to measurements carried out on a two-axis calibration system using a previously described measurement and analysis protocol. As well as imperfections in the calibration system, we simulated imperfect orthogonality of the accelerometer axes and non-identical sensitivity of the individual accelerometers in an otherwise perfect triaxial accelerometer, but we left characterization of other accelerometer imperfections such as non-linearity for future study. Within this framework, sensitivity-matrix errors are caused by imperfections in the construction and installation of the accelerometer calibration system, but not by the accelerometer imperfections included in the simulations. We use the results of this study to assign type B uncertainties to the components of the sensitivity matrix and related intrinsic properties due to imperfections in the measurement system. For calibrations using a reasonably well manufactured and installed multi-axis rotation stage such as that studied in this paper, we estimated upper bounds to the standard uncertainties of the order of 1×10−5, 2×10−5, 5×10−5, and 2×10−4 for the intrinsic sensitivities, diagonal elements of the sensitivity matrix, off-diagonal elements of the sensitivity matrix, and zero-acceleration offsets, relative to a sensitivity-matrix element of 1, respectively, and 5×10−3 degrees for the intrinsic angles.

## 1 Introduction

Very low cost accelerometers produced with microelectromechanical-systems technology (MEMS) have expanded the application of these devices for use in electronic games, smart phones, drones, automobiles, *etc*. At the same time economies of scale and continued technology improvements are producing ever increasing performance requirements for more demanding applications. This provides strong motivation for improving calibration methods for such accelerometers.

There are many measurement and data-analysis protocols for calibrating single-axis, two-axis and three-axis (triaxial) accelerometers with gravity, so the basic ideas are well known and only a separate review article could adequately cite all of the relevant work. The rotations required by these protocols range from a small number carried out with equipment as simple as a hollow cube that can be supported on any of its six sides to those available from accurately calibrated three-axis gimbals. The analysis methods range from sums and differences of measured values to sophisticated vector, matrix, or quaternion mathematics, as well as combinations thereof.

For maximum accuracy, the result of a measurement/analysis protocol of a single accelerometer is reported as a sensitivity vector. The sensitivity vector depends upon the orientation of the accelerometer in a specified coordinate system. The dot product of the sensitivity vector with a vector that quantifies the acceleration experienced by the accelerometer describes the response of the accelerometer to the acceleration. In the case of a triaxial accelerometer, the vectors describing the response of each accelerometer can be measured simultaneously, and these can be combined into an orientation-dependent sensitivity matrix. It is often desirable to decompose the orientation-dependent sensitivity matrix into an orientation-independent set of parameters.

Our choice of analysis protocol [1, 2], which we call the ABC sine-fitting protocol,^1^1Our measurement protocol rotates the accelerometer under test around a single axis in three separate experiments, which enables each experiment to be modeled as a linear, single-harmonic function of a single rotation angle with three adjustable parameters (A, B, C) rather than a nonlinear function of two rotation angles. was motivated by two somewhat conflicting desires. First, we want it to be mathematically simple so that it is easily accessible. However, we also wanted it to be capable of producing mathematical precision limited only by the lesser of the number of bits in the response data of the accelerometer under test (AUT) or the number of bits available to the computer arithmetic. Our choice of measurement protocol was dictated by our choice of analysis protocol, but any other suitable measurement and analysis protocol could have been used.

Our choice of an orientation-independent decomposition for the sensitivity matrix, which we call the intrinsic properties decomposition, was similarly motivated. The intrinsic properties consist of the sensitivity of each accelerometer along its axis of maximum sensitivity, the angle between each pair of axes of maximum sensitivity, and the zero-acceleration response of each accelerometer. These properties provide a complete description of the performance of a linear, drift-free, triaxial accelerometer for a fixed set of pertinent environmental parameters.

We propose the intrinsic properties as a supplement to the sensitivity matrix in comparisons of measurements within and between organizations. A calibration laboratory can use the intrinsic properties for quality control and accelerometer diagnostics. The intrinsic properties can serve as important measurement metrics in comparisons conducted by multiple calibration laboratories. Use of the intrinsic properties rather than the sensitivity matrix to characterize a triaxial accelerometer effectively separates the description of the device from the description of its orientation in a specific application. However, it does not replace the sensitivity matrix. Instead, the intrinsic properties quantify how well different sensitivity-matrix measurements characterize an accelerometer independent of how well the specified orientation of the accelerometer is implemented in each measurement.

Compared to the results of other mathematics-based decompositions, which all have advantages in certain domains, the intrinsic properties are directly interpretable in terms of the physical properties of the device. For instance, attaching a surface-mount MEMS device to a substrate or package can strain the MEMS device. This built-in strain can change over time due to temperature changes or die-attach relaxation and change the intrinsic properties in various ways over time. Not only does an intrinsic-properties decomposition allow alignments errors to be distinguished from sensitivity changes, but it makes it immediately apparent what is changing and by how much.

The version of the ABC protocol that we studied here was optimized for use with a new two-axis AUT calibration system (ACS) for which its manufacturer specifies an angle-setting resolution and accuracy of 0.001° and 0.008°, respectively. During implementation of the protocol, the response of each single-axis

accelerometer within the AUT was measured at *N* uniformly spaced rotation angles for each of three different initial configurations of the ACS. At each angle, a fixed number of measurements were carried out after waiting a sufficient time for the ACS platter to settle. The data obtained at each angle were averaged and analyzed following the analysis portion of the protocol to produce the DC (zero-frequency) components of the sensitivity matrix and intrinsic property triple of the AUT in its default orientation, which is defined below. Separate measurements are required to determine the relative frequency dependence of these quantities if that information is needed [3].

The purpose of this report is to describe the results of simulations of errors in the sensitivity matrix and intrinsic properties caused by imperfections in the alignment and orientation of the AUT and ACS axes during rotation measurements and analysis when following the procedures of the ABC protocol, and ultimately to develop a comprehensive type B uncertainty analysis of the results of the measurements.

## 2 Definition of Simulation Parameters

### 2.1 Local Gravitational Coordinate System, Gravitational Unit ***g*_233_** and Acceleration Vector

Let

$\hat{x}=\left[\begin{array}{l} 1\\ 0\\ 0 \end{array}\right],\hat{y}=\left[\begin{array}{l} 0\\ 1\\ 0 \end{array}\right],\text{ and }\hat{z}=\left[\begin{array}{l} 0\\ 0\\ 1 \end{array}\right],$ (1)

be the local orthonormal gravitation coordinate system (LGCS). Its z-axis points away from the ground and is parallel to the local gravitation field at the center of the top surface of the ACS measurement platter (platform for mounting an AUT). This serves as a reference point.

We define a horizontal surface to be one that is perpendicular to *$\hat{z}$*. Other than orthogonality to *$\hat{z}$* and each other, the orientations of $\hat{x}$ and $\hat{y}$ are not set in advance and can be rotated around *$\hat{z}$* to fit application-specific constraints.

Our ACS is located on the ground floor of the Sound Building (Building 233) on the National Institute of Standards and Technology (NIST) Gaithersburg, MD campus, and the magnitude of the acceleration due to gravity in the vicinity of the ACS is *g*_233_ = 9.80101 ±0.00003 m s^-2^ (k = 2) [4], which provides traceability to SI units [5] for our measurements. To simplify the equations, we use g_233_ as the unit of gravitational acceleration in the following analysis.

Similarly, we use R as a generic unit of accelerometer response because an accelerometer output may be specified as a voltage, a charge, a current, a unitless number, a resistance, or a capacitance, or it can be scaled to be read as meters per second squared (m s^-2^) or gravitational units "(g). Thus, for the purposes of this paper, an accurately calibrated accelerometer reports 1 R when excited by an acceleration of 1 g_233_ along its axis of maximum sensitivity and the default sensitivity matrix of an ideal AUT perfectly mounted on an ideal ACS is the unit matrix ***I*** multiplied by R/g_233_.

Next, let

$a(n)=\left[\begin{array}{l} a_{x}(n)\\ a_{y}(n)\\ a_{z}(n) \end{array}\right]$ (2) 

for *n* = 1,..., *N* rotation angles, where the x, y, and z components indicate the acceleration experienced by an AUT during a series of measurements carried out at different angular orientations of the ACS platter.

We start with *n* = 1 rather than *n* = 0 and reserve *n* = 0 for the default orientation of the ACS axes and platter when the instrument is first activated or reset. Therefore the reference angle from which rotations around the two ACS axes are measured are both zero when *n* = 0. To start a rotation experiment, *n* is set to 1 and one or both of the axes are rotated to specified initial orientations for the desired experiment and the data indexed by *n* = 1 are recorded.

### 2.2 Accelerometer Calibration System (ACS), Rotation Axes $\hat{X}$ and $\hat{Z}$, and Rotations **Θ*_X_* (*n*)** and **Θ*_Z_*(*n*)**

In its most abstract but still complete representation, our ACS comprises

⏺ two approximately orthogonal rotation axes, $\hat{X}$ and $\hat{Z}$, the latter of which is configured to rotate around $\hat{X}$, 

⏺ a means for mounting a device under test in a fixed orientation relative to $\hat{Z}$, and

⏺ a means for rotating each axis through a set of user-specified angles.

Prior to a rotation experiment, $\hat{X}$ and $\hat{Z}$ are rotated through angle Θ*_X_* (1) and angle Θ*_Z_*(1), respectively, to transform the ACS from its default configuration to the initial configuration for the experiment. During the rotation experiments simulated here, the accelerometer response as a function of rotation angle Θ*_X_* (*n*) or Θ*_Z_*(*n*) is measured for *n* = 1,..., *N* rotation angles while the other angle is held constant.

**Rotation-independent errors:** Ideally, when the ACS is installed in our laboratory, $\hat{X}$ is collinear with $\hat{x}$ and $\hat{Z}$is collinear with $\hat{z}$. In practice this is only an approximation that must be modeled in the simulation.

In the default configuration of the ACS as installed in our laboratory, $\hat{Z}$ is given by

$\hat{Z}=\left[\begin{array}{l} {\hat{Z}}_{X}\\ {\hat{Z}}_{y}\\ \sqrt{1-\left({\hat{Z}}_{X}^{2}+{\hat{Z}}_{y}^{2}\right)} \end{array}\right]=RRF\left(\hat{z},\hat{E},\Theta_{E}\right),$ (3)

where *RRF*(*$\overset{➝}{d},\hat{e},\alpha$*) is the Rodrigues rotation formula [6] that rotates a column (row) vector $\overset{➝}{d}$ around a unit vector $\hat{e}$ by an angle *α* and returns the result as a column (row) vector.

Euler's rotation theorem guarantees that the unit vector $\hat{E}$ and the angle Θ*_E_* in Eq. (3) both exist, but they are not unique. A right-hand (left-hand) rotation Θ*_E_* around the $\hat{E}$ axis produces the same result as a right-hand (left-hand) rotation -Θ*_E_* around the -$\hat{E}$ axis.

Similar to $\hat{Z}$ in Eq. (3), $\hat{X}$ is given in terms of the LGCS as

$\hat{X}=\left[\begin{array}{l} \sqrt{1-\left({\hat{X}}_{y}^{2}+{\hat{X}}_{z}^{2}\right)}\\ {\hat{X}}_{y}\\ {\hat{X}}_{z} \end{array}\right]=RRF\left(\hat{x},\hat{E},\Theta_{E}\right).$ (4)

It is convenient at this point to use the rotational degree of freedom of the LGCS to rotate the LGCS around $\hat{z}$ to set ${\hat{X}}_{y}$ = 0. With this final constraint on the LGCS, the ACS-parameter set {${\hat{Z}}_{X},{\hat{Z}}_{y},{\hat{X}}_{z}$} comprises a maximum-size set of independent scalers that define the default configuration of the ACS in our laboratory relative to the LGCS.

It might appear that it would be more convenient to use {*E_x_*, *E_y_*,Θ*_E_* } as the ACS alignment parameters. However, the combination of two projections and an angle, and the fact that $\hat{E}$ and Θ*_E_* are not unique would greatly complicate the interpretation of experiments designed to determine their values directly from the experiments rather than determining them from the values of {${\hat{Z}}_{X},{\hat{Z}}_{y},\text{and }{\hat{X}}_{z}$} obtained from the same experiments.

We separately studied the effect of each of ${\hat{Z}}_{X},{\hat{Z}}_{y},{\hat{X}}_{z}$ on the sensitivity matrix in an attempt to find interesting or useful properties and the results are reported in Appendix A. Only one useful property was discovered. The magnitude of the largest error (dominant error) in the sensitivity matrix and other errors of approximately the same magnitude scaled with the magnitude of the alignment error being studied over a range of alignment errors extending from sin(0.0008°) to sin(0.08°). Other errors were orders of magnitude smaller with the details dependent upon the particular alignment error being studied.

From a physical point of view, the ACS has a rod whose axis of rotation coincides with $\hat{Z}$ and is free to rotate around $\hat{Z}$. A platter is connected to the rod such that the platter's top surface is nominally perpendicular to $\hat{Z}$. However, from the abstract point of view, it is much more convenient to treat the platter and its misalignment relative to $\hat{Z}$as part of the misalignment associated with mounting the AUT on the ACS as described later in this report.

**Rotation-step errors:** Ideally, when the ACS executes a rotation step, the step is exactly the size desired. In practice this is only an approximation that must be modeled in the simulation. Therefore, we generalize each rotation angle in each rotation experiment as

Θ*_J_*(*n*) = Θ*^i^* . . . *J*(*n*) = *α*(*n*) +$f_{J}^{i}$(*n*), (5)

where *i* ∈ {1, 2, 3} indexes the rotation experiment number, *J* ∈ {*X*, *Z*} (not to be confused with *j* ∈ {*u*, *v*, *w*}) indexes the rotation axis, *n* ∈ {1,..., *N*} indexes the rotation step, *α*(*n*) is the desired rotation angle, and $f_{J}^{i}$(*n*) is the rotation-step error for the *n^th^* rotation step. Note that the superscript *i* of $\Theta_{J}^{i}$(*n*), which indexes the rotation experiments, will be suppressed when it is superfluous to the point being made, or is obvious from context.

### 2.3 AUT, Sensitivity Matrix, and Intrinsic Property Triple

Assume that the AUT comprises U, V, and W accelerometers each having unique axes of maximum sensitivity $\hat{u},\hat{v},\text{and }\hat{w}$ that do not need to be perfectly orthogonal to each other. Therefore, at each rotation measurement step *n* = 1,..., *N* with the AUT securely mounted on the ACS platter,

$\hat{u}(n)=\left[u_{x}(n)\;\;u_{y}(n)\;\;u_{z}(n)\right]=\;\left[\sqrt{1-u_{y}^{2}(n)-u_{z}^{2}(n)\;\;}u_{y}(n)\;\;u_{z}(n)\right],$

$\hat{v}(n)\;\;=\left[v_{x}(n)\;\;{\text{ v}}_{y}(n)\;\;{\text{ v}}_{z}(n)\;\;\right]=\left[v_{x}(n)\sqrt{1-v_{x}^{2}(n)-v_{z}^{2}(n)\;\;}\;\;v_{z}(n)\right],$ (6)

$\hat{w}(n)=\left[w_{X}(n)\;\;w_{y}(n)\;\;w_{z}(n)\right]=\;\;\left[w_{x}(n)\;\;w_{y}(n)\;\;\sqrt{1-w_{x}^{2}(n)-w_{y}^{2}(n)}\right].$

Note that the constraints explicit in the right-hand sides of these equations assure that $\hat{u}$(*n*), $\hat{v}$(*n*), and $\hat{w}$(*n*) are unit vectors.

Further note that even though $\hat{u}$(*n*), $\hat{v}$(*n*), and $\hat{w}$(*n*) vary with *n*, the dot products $\hat{u}$(*n*)· $\hat{v}$(*n*), $\hat{v}$(*n*) · $\hat{w}$(*n*), and $\hat{w}$(*n*) · $\hat{u}$(*n*), as well as the angles they define are independent of the orientation of the AUT and therefore are independent of *n* for a rigid AUT. Therefore, the angles, which are explicitly defined below, define three intrinsic properties of the AUT.

Assume that the response ***r*** of each of the triaxial accelerometers in the AUT is a linear function of the acceleration that they experience. In this case, the response during the *n^th^* response measurement in a measurement series will be given by

$r(n)=\left[\begin{array}{l} r_{u}(n)\\ r_{v}(n)\\ r_{w}(n) \end{array}\right]=s(n)\alpha (n)+O\text{=}\left[\begin{array}{l} s_{ux}(n)\;\;s_{uy}(n)\;\;s_{uz}(n)\\ s_{vx}(n)\;\;s_{vy}(n)\;\;s_{vz}(n)\\ s_{wx}(n)\;\;s_{wy}(n)\;\;s_{wz}(n) \end{array}\right]\left[\begin{array}{l} a_{x}\left(n\right)\\ a_{y}\left(n\right)\\ a_{z}\left(n\right) \end{array}\right]+\left[\begin{array}{l} O_{u}\\ O_{v}\\ O_{w} \end{array}\right],$ (7)

where ***s***(*n*) is the sensitivity matrix and ***O*** is the zero-excitation offset triple, which describes the response of the U, V, and W accelerometers when *a_x_* = *a_y_* = *a_z_* = 0. As mentioned previously, the default sensitivity matrix of an ideal AUT perfectly mounted on a perfectly installed ACS would be the unit matrix ***I ***multiplied by R/g_233_. Note that ***O*** is independent of the orientation of the accelerometers, so it defines three intrinsic properties, and it is called the intrinsic offset triple or just the offset triple. The sensitivity matrix in Eq. (7) can be expressed in a number of ways,

$s(n)=\left[\begin{array}{l} s_{u}u_{x}(n)\;\;s_{u}u_{y}(n)\;\;s_{u}u_{z}(n)\\ s_{v}v_{x}(n)\;\;s_{v}v_{y}(n)\;\;s_{v}v_{z}(n)\\ s_{w}w_{x}(n)\;\;s_{w}w_{y}(n)\;\;s_{w}w_{z}(n) \end{array}\right]=\left[\begin{array}{l} s_{ux}(n)\;\;s_{uy}(n)\;\;s_{uz}(n)\;\;\\ s_{vx}(n)\;\;s_{vy}(n)\;\;s_{vz}(n)\\ s_{wx}(n)\;\;s_{wy}(n)\;\;s_{wz}(n) \end{array}\right]=\left[\begin{array}{l} {\overset{➝}{s}}_{u}(n)\\ {\overset{➝}{s}}_{v}(n)\\ {\overset{➝}{s}}_{w}(n) \end{array}\right],$ (8)

where the intrinsic sensitivity matrix

$s=\left[\begin{array}{l} s_{u}\\ s_{v}\\ s_{w} \end{array}\right]=\left[\begin{array}{l} \left||{\overset{➝}{s}}_{u}(n)|\right|\\ \left||{\overset{➝}{s}}_{v}(n)|\right|\\ \left||{\overset{➝}{s}}_{w}(n)|\right| \end{array}\right]$ for any n=1,...,*N *(9)

gives the response of the U, V, and W accelerometers per unit acceleration applied along the $\hat{u}$, $\hat{v}$, and $\hat{w}$ axes after subtraction of *O_x_*, *O_y_*, and *O_z_*, respectively, and ‖...‖ denotes the magnitude of the vector between the bars. Note that the intrinsic sensitivity is independent of *n* so its three components are intrinsic properties.

It is important to distinguish between the sensitivity matrix ***s***(*n*) with its sensitivity-vector components ${\overset{➝}{s}}_{u}\left(n\right),{\overset{➝}{s}}_{v}\left(n\right),and{\overset{➝}{s}}_{w}\left(n\right)$ on one hand, and the intrinsic sensitivity triple **s** with its components, **s***_u_*, **s***_v_*, and **s***_w _*on the other hand. This is why we use a different typeface for the latter.

Note also that ***a*** = 0 when an accelerometer is accelerating toward the Earth in free fall and that ***a*** = *g*_ℓ_*_oc_$\hat{z}$* relative to the LGCS at the location of the accelerometer when it is at rest at some fixed location on Earth, where *g*_ℓ_*_oc_* is the magnitude of the acceleration due to gravity at that location. Though counterintuitive, this convention is consistent with the general theory of relativity and describes the output of most accelerometers because they actually respond to deflection of a structural element attached to a rigid package. However, some accelerometers, such as piezoelectric ones and those with high-pass filters, have no DC response, so the protocol as described here is not applicable to that type of accelerometer.

The sensitivity matrices depend upon the *n*-dependence of *u_y_*(*n*), *u_z_*(*n*), *v_x_*(*n*), *v_z_*(*n*), *w_x_*(*n*), and *w_y_*(*n*), but these dependencies are not errors. Instead they are necessary to describe the orientation of the AUT accelerometers relative to the LGCS as the AUT is rotated. It is the orientation of these axes relative to the LGCS when *n* = 0 that determines the default sensitivity matrix relative to the LGCS.

Besides the intrinsic-offset triple ***O*** and the intrinsic-sensitivity triple **s**, the intrinsic-angle triple mentioned above [angle between the $\hat{u}$(*n*) and $\hat{v}$(*n*), $\hat{v}$(*n*) and $\hat{w}$(*n*), and $\hat{w}$(*n*) and $\hat{u}$(*n*)] axes, respectively), is also an intrinsic property triple given by

$ⲫ=\left[\begin{array}{l} {ⲫ}_{uv}\\ {ⲫ}_{vw}\\ {ⲫ}_{wv} \end{array}\right]=\left[\begin{array}{l} \text{arcos }(\hat{u}(n)\cdot \hat{v}(n))\\ \text{arcos }(\hat{v}(n)\cdot \hat{w}(n))\\ \text{arcos }(\hat{w}(n)\cdot \hat{u}(n)) \end{array}\right]=\left[\begin{array}{l} \text{arcos }(\hat{u}(0)\cdot \hat{v}(0))\\ \text{arcos }(\hat{v}(0)\cdot \hat{w}(0))\\ \text{arcos }(\hat{w}(0)\cdot \hat{u}(0)) \end{array}\right]$, (10)

independent of *n*. 

### 2.4 Rotation Experiment Equations

Recall that we defined the default orientation of the AUT as its orientation on the ACS following the mounting specifications provided by the manufacturer of the AUT after the ACS has been turned on. We define the default sensitivity matrix of the AUT as the sensitivity matrix that describes the response of the AUT in its default orientation on the ACS.

Assume that the ACS is in its default configuration in our laboratory and that the AUT device is firmly affixed to the ACS platter such that $\hat{u}$(*n* = 0) = $\hat{v}$(*n* = 0) = $\hat{w}$(*n* = 0) = 0 are approximately parallel to $\hat{x}$, $\hat{y}$, and $\hat{z}$ of the LGCS, respectively. At this point, the ACS can be rotated from the default configuration to the desired initial configuration for a measurement of the accelerometer response versus rotation angle, and the measurements series can commence.

The measurement protocol described in [2] requires the response of the AUT to be measured in three rotation experiments: 1) as a function of rotation-step number *n* around $\hat{X}$, 2) as a function of *n* around $\hat{X}$ after the AUT has first been rotated through an angle Θ*_Z_* = -90° around $\hat{Z}$, and 3) as a function of *n* around $\hat{Z}$ after the AUT has first been rotated through an angle Θ*_X_* = -90° around $\hat{X}$ .

With a real AUT mounted on a real ACS, our implementation of rotation experiment 1 with the ABC protocol is given in terms of the Rodrigues rotation formula (RRF) defined following E q. (3) as $\begin{array}{l} r_{1}(\alpha )=\left[\begin{array}{l} r_{1u}(\alpha (n))\\ r_{1v}(\alpha (n))\\ r_{1w}(\alpha (n)) \end{array}\right]=\left[\begin{array}{l} {\overset{➝}{s}}_{u}(n)\cdot g_{233}\hat{z}+Ou\\ {\overset{➝}{s}}_{v}(n)\cdot g_{233}\hat{z}+Ou\\ {\overset{➝}{s}}_{w}(n)\cdot g_{233}\hat{z}+Ou \end{array}\right]=\left[\begin{array}{l} RRF({\overset{➝}{s}}_{u}(1),\hat{X},\alpha (n))\cdot g_{233}\hat{z}+Ou\\ RRF({\overset{➝}{s}}_{v}(1),\hat{X},\alpha (n))\cdot g_{233}\hat{z}+Ov\\ RRF({\overset{➝}{s}}_{w}(1),\hat{X},\alpha (n))\cdot g_{233}\hat{z}+Ow \end{array}\right], \end{array}$ (11)

where Θ*_X_* (1) = Θ*_Z_*(1) = 0° for both the default configureation and the initial configuration of the ACS in this experiment, and it is convenient to use *α*(*n*) = Θ*_X_* (*n*) for the term called *α_i_* in [2], as well as to clearly distinguish between the initial rotation and the rotations carried out during the measurement process.

For experiment 2, our implementation is given by

$r_{2}(\alpha )=\left[\begin{array}{l} r_{2u}(\alpha (n))\\ r_{2v}(\alpha (n))\\ r_{2w}(\alpha (n)) \end{array}\right]=\left[\begin{array}{l} RRF(RRF({\overset{➝}{s}}_{u}(1),\hat{Z},\Theta_{Z}(1)),\hat{X},\alpha (n))\cdot g_{233}\hat{z}+Ou\\ RRF(RRF({\overset{➝}{s}}_{v}(1),\hat{Z},\Theta_{Z}(1)),\hat{X},\alpha (n))\cdot g_{233}\hat{z}+Ov\\ RRF(RRF({\overset{➝}{s}}_{w}(1),\hat{Z},\Theta_{Z}(1)),\hat{X},\alpha (n))\cdot g_{233}\hat{z}+Ov \end{array}\right].$ (12)

In this case, *α*(*n*) still represents Θ*_X_* (*n*), but Θ*_Z_*(1) = -90° to transform the default configuration of the ACS to the initial configuration for experiment 2.

For experiment 3, our implementation is given by,

$r_{3}(\alpha )=\left[\begin{array}{l} r_{3u}(\alpha (n))\\ r_{3v}(\alpha (n))\\ r_{3w}(\alpha (n)) \end{array}\right]=\left[\begin{array}{l} RRF(RRF({\overset{➝}{s}}_{u}(1),\hat{X},\Theta_{X}(1)),RRF((\hat{Z},\hat{X},\Theta_{X})\alpha (n))\cdot g_{233}\hat{z}+Ou\\ RRF(RRF({\overset{➝}{s}}_{v}(1),\hat{X},\Theta_{X}(1)),RRF((\hat{Z},\hat{X},\Theta_{X}),\alpha (n))\cdot g_{233}\hat{z}+Ov\\ RRF(RRF({\overset{➝}{s}}_{w}(1),\hat{X},\Theta_{X}(1)),RRF((\hat{Z},\hat{X},\Theta_{X}),\alpha (n))\cdot g_{233}\hat{z}+Ow \end{array}\right].$ (13)

## 3 Simulation of ABC Rotation Experiments Without Rotation-Angle Errors

Simulation Set 1 simulated use of the ABC analysis protocol to analyze response data from an ideal triaxial AUT when measured with an ideal ACS following the ABC measurement protocol.

A MATLAB^1^ [7] simulation script was used to calculate the response versus rotation angle data *r_ij_*(*α*(*n*)) of the AUT accelerometers for rotation experiments *i* ∈ {1, 2, 3} and accelerometers *j* ∈ {*u*, *v*, *w*} with ***s*** = ***I***, where ***I*** is the unit matrix, and ***O*** = 0, as in Eqs. (11-13).

**Fig. 1:**
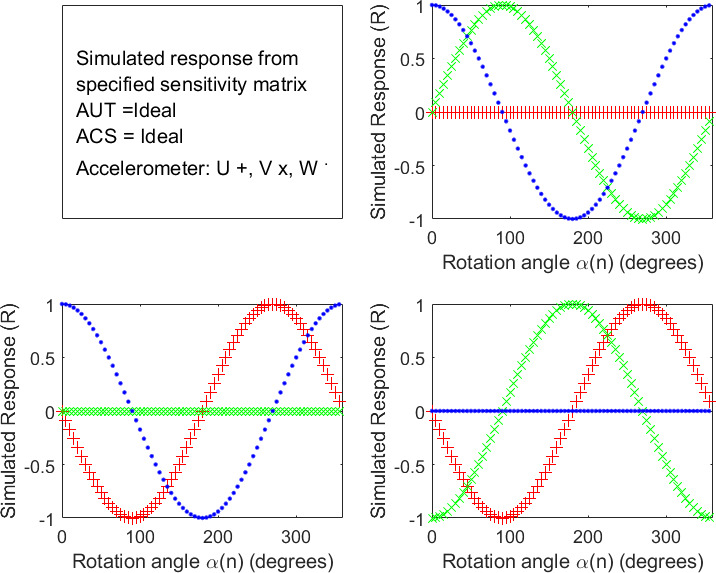
Simulated accelerometer response data versus rotation angle for an ideal AUT measured on an ideal ACS in rotation experiments 1 (top-right panel), 2 (lower-left panel), and 3 (lower-right panel), where R is the generic response unit corresponding to an acceleration of 1 g_233_. (Accelerometer response symbols: U red +, V green x, W blue _._)

The ACS properties are summarized in Table 1. More specifically, the desired rotation-step size is Δ = 5^°^, *α*(*n*) = (*n* - 1)Δ for *n* = 1,..., *N* = 72, ${\hat{Z}}_{X},{\hat{Z}}_{y},\text{and }{\hat{X}}_{z}$ = 0, and the other parameters in Eqs. (11-13) were calculated with Eqs. (3) and (4). The simulated response data are shown in Fig. 1.

**Table 1:** ACS parameters used in Simulation Set 1 which assumed an ideal AUT and a perfectly constructed and installed ACS.

ACS Parameters					
	Alignment			Rotation	
Experiment	${\hat{Z}}_{X}$	$\hat{Z}$ *_y_*	${\hat{X}}_{z}$	$\Theta_{X}(n)$	$\Theta_{z}(n)$
1	0	0	0	*α*(*n*)	0°
2	0	0	0	*α*(*n*)	-90°
3	0	0	0	-90°	*α*(*n*)

**Fig. 2:**
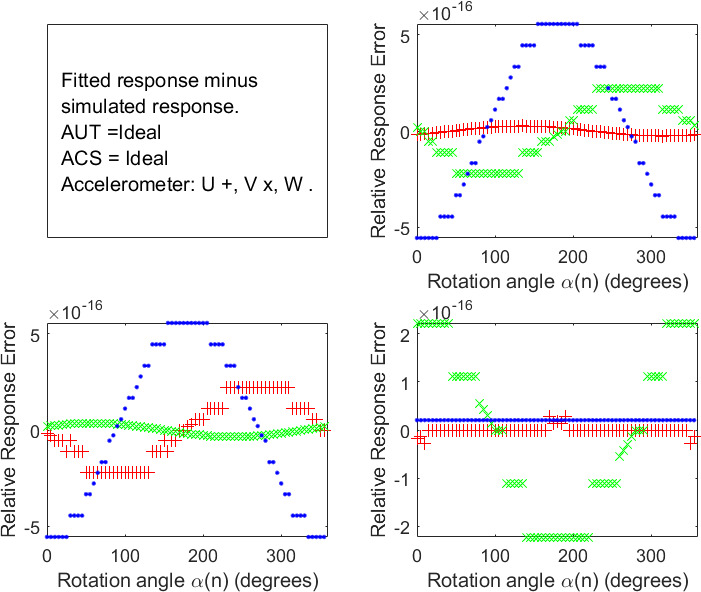
Difference between the simulated accelerometer response data versus rotation angle shown in Fig. 1 and that obtained as described in the text from an ABC fit to the simulated data. The results for rotation experiments 1, 2, and 3 are shown in the upper right, lower left, and lower right panels, respectively. (Accelerometer response symbols: U red +, V green x, W blue _._)

The simulation script then wrote the response versus rotation-angle data *r_ij_*(*α*(*n*)) into an Excel[8] spreadsheet that implemented the ABC analysis protocol of [2]. This spreadsheet then calculated estimates ***s****_FIT_* and ***C****_FIT_* of ***s*** and ***O***, respectively, by fitting $A_{jk}^{i}$ sin(Θ) + $B_{jk}^{i}$ cos(Θ) + $C_{j}^{i}$ to *r_ij_*(*α*(*n*)) as described in [2] with the changes in notation given in Appendix B, and where *k* ∈ $\left\{\hat{x},\hat{y},\hat{z}\right\}$.

When this step was complete, the simulation script read ***s****_FIT_* and ***C****_FIT_* from the spreadsheet, calculated the intrinsic property triples,${\overset{➝}{S}}_{\mathrm{FIT}}$ and ***ⲫ****_FIT_*, and calculated the differences ***s****_ERR_* = ***s****_FIT_* - ***s***, ${\overset{➝}{s}}_{\mathrm{ERR}}$ =${\overset{➝}{s}}_{\mathrm{FIT}}$ -$\overset{➝}{s}$, ***ⲫ****_ERR_* = ***ⲫ****_FIT_* - ***ⲫ***, and ***O****_ERR_* = ***C****_FIT_* - ***O***, which are the errors introduced by this implementation of the ABC protocols. The script also calculated a new set of response versus rotation angle data *r_ij_*(*α*(*n*))*__Fit__* from ***s****_FIT_* and ***C****_FIT_*, which is plotted in Fig. 2 for comparison with the original simulated response data *r_ij_*(*α*(*n*)) in Fig. 1.

No values of the components of ***s****_ERR_*, ${\overset{➝}{s}}_{\mathrm{ERR}}$, and ***O****_ERR_* fell outside ±10^-15^, and no values of the intrinsic-angle error ***ⲫ****_ERR_* fell outside ±10^-13^degrees. These errors are completely explained by round-off errors in computer arithmetic and algorithms. This result is important because it shows that the script and spreadsheet are working properly and sets a lower bound on the uncertainty of the ABC protocol as implemented in [2].

**Simulation Set 2** comprised 30 independent simulations of rotation experiments 1, 2, and 3 using the same ideal ACS properties that were used for Simulation Set 1 (Table 1) but with randomly generated non-ideal AUT parameters. The goal was to isolate the effect of non-ideal AUT parameters on the ABC measurement and analysis protocol. For each of the 30 simulations, the diagonal elements of the default sensitivity matrix ***s*** of the AUT were independently drawn from a normal distribution with a mean of 1 and standard deviation of 0.1 R/g_233_. Similarly, the three values of the offset matrix ***O*** were drawn from a normal distribution with a mean of zero and a standard deviation of 0.1 R. These unrealistically large deviations from the ideal unit sensitivity matrix and zero offset matrix, were chosen to stress the ABC protocols.

The default sensitivity matrix ***s*** and AUT offset matrix *O* for the first simulation in Simulation Set 2 and the intrinsic properties triples calculated from Eq. (9) are given in Table 2. By chance, this sensitivity matrix is the least perfect matrix produced by the 30 simulations.

**Table 2:** One of 30 random sensitivity matrices and the associated intrinsic properties triples. In the array format used here, the vectors are row arrays and the triples are column arrays displayed in a row format in the table.

Randomly Generated Sensitivity Matrix and Associated Intrinsic Properties Triples					
Matrix	Vector	*x* Component	*y* Component	*z* Component	Unit
	${\vec{S}}_{u}$	+1.084557444843417	-0.047097516966133	+0.037216463393415	R/g_233_
***s***	${\vec{S}}_{v}$	+0.369246379560599	+0.920196068359764	+0.285771705579544	R/g_233_
	${\vec{S}}_{w}$	-0.117336380485872	+0.263805007662141	+0.819879210182046	R/g_233_
	Triple	*u* Component	*v* Component	*w* Component	Unit
	**s**	+1.086217332036812	+1.031876523985898	+0.869231170369473	R/g_233_
	***O***	+0.053766713954610	+0.183388501459509	-0.225884686100365	R
	***ⲫ***	+70.845583191199623	+61.081782813884068	+96.639644038935558	Degrees

**Fig. 3:**
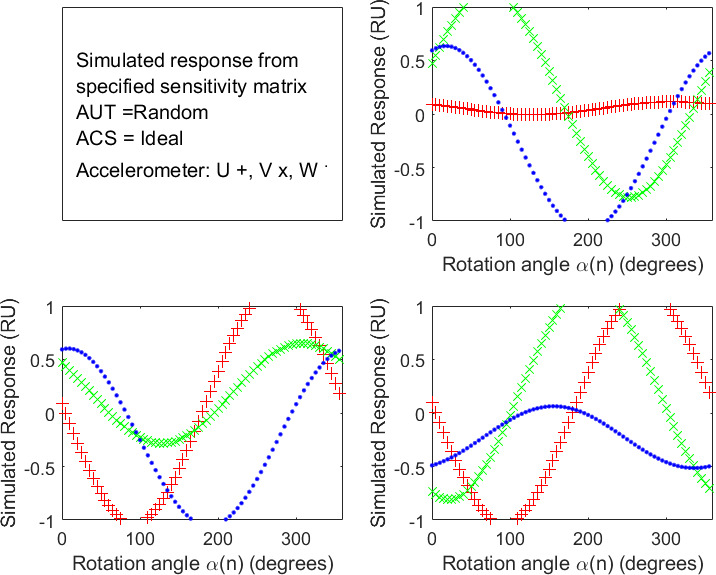
Simulated accelerometer response data versus rotation angle for the first simulation of a stable, linear AUT with random variations from the ideal unit sensitivity matrix of Simulation Set 2 when measured on an ideal ACS in Rotation Experiments 1 (top-right panel), 2 (lower-left panel), and 3 (lower-rights panel), where R is the generic response unit corresponding to an acceleration of 1 g_233_. (Accelerometer response symbols: U red +, V green x, W blue .)

In each of the 30 simulations, the MATLAB simulation script also calculated the response versus rotation-angle data *r_ij_*(*α*(*n*)) of the AUT accelerometers for rotation experiments *i* ∈ {1, 2, 3} and accelerometers *j* ∈ {*u*, *v*, *w*}. The response versus rotation-angle data *r_ij_*(*α*(*n*)) for the randomly generated, default sensitivity matrix given in Table 2 are shown in Fig. 3

For each simulation, the simulation script then wrote the response versus rotation-angle data *r_ij_*(*α*(*n*)) into the Excel ABC spreadsheet that implemented the ABC analysis protocol of [2]. This spreadsheet then calculated estimates ***s****_FIT_* and ***C****_FIT_* of ***s*** and ***O***, respectively, by fitting $A_{jk}^{i}$ sin(Θ) +$B_{jk}^{i}$ cos(Θ) +$C_{j}^{i}$ to *r_ij_*(*α*(*n*)) as described in [2] (with the changes in notation given in Appendix B), and where *k* ∈ $\left\{\hat{x},\hat{y},\hat{z}\right\}$.

**Fig. 4:**
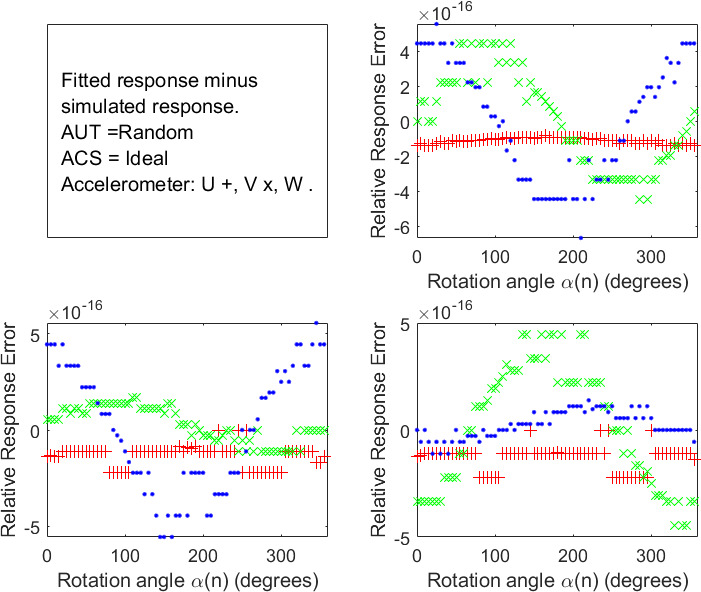
Difference between the simulated accelerometer response data versus rotation angle shown in Fig. 3 and that obtained as described in the text from an ABC fit to the simulated data. The results for Rotation Experiments 1, 2, and 3 are shown in the upper right, lower left, and lower right panels, respectively. (Accelerometer response symbols: U red +, V green x, W blue .)

When this step was complete, the simulation script read ***s****_FIT_* and ***C****_FIT_* from the spreadsheet, calculated the intrinsic property triples, ${\overset{➝}{S}}_{\mathrm{FIT}}$ and ***ⲫ****_FIT_*, and calculated the differences ***s****_ERR_* = ***s****_FIT_* - ***s***, ${\overset{➝}{s}}_{\mathrm{ERR}}$ =${\overset{➝}{s}}_{\mathrm{FIT}}$ -$\overset{➝}{s}$, ***ⲫ****_ERR_* = ***ⲫ****_FIT_* - ***ⲫ***, and ***O****_ERR_* = ***C****_FIT_* - ***O***, which are the errors introduced by this implementation of the ABC protocols. The script also calculated and plotted a new set of response versus rotation-angle data *r_ij_*(*α*(*n*)) *__Fit__* from ***s****_FIT_* and ***C****_FIT_* (shown in Fig. 4) for comparison with *r_ij_*(*α*(*n*)) *__Fit__*in Fig. 3.

For all 30 simulations, the maximum errors s*_ERR_*,${\overset{➝}{s}}_{\mathrm{ERR}}$, and ***O****_ERR_* never fell outside ±10^-15^, and the maximum angular error ***ⲫ****_ERR_* never fell outside ±10^-13^degrees. While this result was expected based on Ref. [1] and Ref. [2], it is nevertheless an important affirmation of the conclusions reported in those references. Differences significantly greater than those observed would show there was a fatal error in the derivations in Ref. [1], an error in the implementation of the ABC measurement and analysis in Ref. [2], or an error in the simulation script.

Also, for each simulation in Simulation Set 2, the spread sheet carried out a second sine fit that included a constant term and the first five harmonics (10 adjustable parameters) of a 360° rotation for the three rotation experiments in the 30*^th^* simulation. All of the coefficients for all of the harmonics greater than the first fell within ±10^-15^. This is important because it means that the ABC coefficients derived in the ABC protocol from fits of the constant C and the first harmonics of a 360° rotation (A and B or magnitude and phase) are sufficient to describe any perfectly stable AUT to within ±10^-15^ over a range of accelerations where the AUT response is linear within the precision of the computer arithmetic.

Within the framework of these simulations, we demonstrated that the ABC protocol reproduces the default sensitivity matrix and intrinsic properties triples of triaxial AUTs with non-orthogonal axes and non-identical gains when the rotation experiments are carried out with a perfect ACS. Now we use the same framework to simulate the effect of an imperfect ACS.

## 4 Simulation of Rotation Experiments on an Imperfect AUT with an Imperfect ACS

Ideally, we would have measured values and standard uncertainties for all of the ACS errors identified in Sec. 2.2. In that case we would use these uncertainties as the basis for estimating type A uncertainties in the elements of the sensitivity matrix associated with use of the ABC protocol. Unfortunately, that is not the case as explained at the end of Appendix A. Instead, we randomly varied all of these errors with a standard deviation equivalent to the ACS manufacturer's stated uncertainty in a Monte Carlo simulation and used the standard deviation of the resulting default sensitivity matrices and intrinsic properties to assign type B uncertainties.

**Simulation Set ABC1**: For each of 1000 simulations, the simulation procedure described for Simulation Set 2 was followed with one major exception. The diagonal elements of the default sensitivity matrix ***s*** of the AUT independently were drawn from a normal distribution with a mean of 1 and a standard deviation 0.1 R/g_233_, and the off-diagonal elements of the default sensitivity matrix ***s*** independently drawn from a normal distribution with a mean of 0 and the same standard deviation. In addition, the values of ${\hat{Z}}_{X},{\hat{Z}}_{y},\text{ and }{\hat{X}}_{z}$ were drawn from a normal distribution with a mean of 0 and a standard deviation of sin(0.008°) = 1.3963 × 10^-4^, and the values of $f_{j}^{i}(n)$ [defined in Eq. (5)] for each of *N* = 72 values of *n* were drawn from a normal distribution with a mean of 0 and standard deviation of 0.008°, where 0.008° is the manufacturer-stated accuracy of our ACS. The resulting sensitivity-matrix errors and the intrinsic properties errors that were introduced by the simulated ACS imperfections are shown in Tables 3 and 4, respectively.

**Table 3:** Mean and standard deviation of simulated sensitivity-matrix error of the randomly generated AUT defined in Table 2 and measured with the 1000 independent randomly generated ACS parameters described above.

Means of Errors in Sensitivity Matrix					
Matrix	Vector	*x* Component	*y* Component	*z* Component	Unit
	${\overset{➝}{s}}_{u}$	+2.610e-07	+1.596e-07	+6.213e-07	R/g_233_
***s***	${\overset{➝}{s}}_{v}$	-8.222e-07	+6.825e-08	+8.041e-07	R/g_233_
	${\overset{➝}{s}}_{w}$	-1.624e-06	+6.073e-07	-4.201e-07	R/g_233_
Standard Deviations of Errors in Sensitivity Matrix					
Matrix	Vector	*x* Component	*y* Component	*z* Component	Unit
	${\overset{➝}{s}}_{u}$	+1.709e-05	+7.289e-05	+1.274e-04	R/g_233_
***s***	${\overset{➝}{s}}_{v}$	+7.403e-05	+1.002e-05	+1.195e-04	R
	${\overset{➝}{s}}_{w}$	+1.548e-04	+6.821e-05	+1.760e-05	Degrees

**Table 4:** Means and standard deviations of the intrinsic properties calculated from the 1000 simulations whose means and standard deviations are listed in Table 3.

Mean Errors and Standard Deviations of Intrinsic PropertyTriples			
Intrinsic Property	Mean Error	Std. Dev.	Unit
**s** *_u_*	+7.514e-08	+9.244e-06	R/g_233_
**s** *_v_*	-1.192e-07	+9.609e-06	R/g_233_
**s** *_w_*	+4.152e-07	+1.309e-05	R/g_233_
***O*** *_u_*	-1.677e-07	+4.894e-05	R
***O*** *_v_*	-4.273e-07	+4.892e-05	R
***O*** *_w_*	+1.272e-07	+4.562e-05	R
***ϕ*** *uv*	-4.079e-06	+7.555e-04	Degrees
***ϕ*** *vw*	+1.539e-05	+5.447e-03	R
***ϕ*** *wu*	+2.324e-05	+5.431e-03	R

We carried out another 500 independent simulations (results not shown) with the rotation-step error given by

$f_{j}^{i}(n)=F_{Jm}^{i}+\sum_{m=1}^{N/2}D_{Jm}^{i}\sin(m\alpha (n))+E_{Jm}^{i}\cos(m\alpha (n)),$ (14)

where $D_{Jm}^{i}$and $E_{Jm}^{i}$were drawn from a normal distribution with mean 0 and standard deviation 0.008°.

The results were similar to those shown in Tables 3 and 4 with an important exception. The magnitudes of most of the means in Tables 4 are greater than the corresponding means in Table 3. This is partly explained by the use of 500 simulations for the former and 1000 for the latter. However, none of the means in Table 3 were statistically different from zero at the 95% confidence level when$f_{J}^{i}(n)$ was drawn from a random distribution, but three were statistically different from zero at the 95% confidence level when $D_{Jm}^{i}$and $E_{Jm}^{i}$ were drawn from the same random distribution. This make sense because modeling$f_{J}^{i}(n)$with a low harmonic sine function produces correlations in $f_{J}^{i}(n)$for small regions of *n*. The net effect is to magnify large errors at the peaks of the sine functions and minimize small errors at the zero crossings of the sine function, and these effects increases the large contribution to the variance more than they decrease the small contributions.

**Simulation Set ABC2**: To stress the ABC protocol for synchronous rotation-step errors in Eq. (14), we set ${\hat{Z}}_{X}={\hat{Z}}_{y}\text{=}{\hat{X}}_{z}=0$, set $D_{J1}^{i}=E_{J1}^{i}=6$∗sin(0.008^°^), which far exceeds the manufacturer's accuracy statement, set the remaining Fourier coefficients to zero, and calculated the sensitivity matrix and intrinsic properties errors using the procedures described above. The absolute values of the errors in the components of the sensitivity matrix and intrinsic property triples were never as large as the corresponding standard-deviation uncertainties in Tables 3 and 4.

To further stress the protocol, we then set $-{\hat{X}}_{z}={\hat{Z}}_{x}={\hat{Z}}_{y}$ = $D_{J1}^{i}=E_{J1}^{i}=6$∗ sin(0.008°). This change produced errors with magnitudes equal to 2.1 × 10^-4^ in *s_uz_* and *s_wx_*, which are 1.36 times as large as the largest off-diagonal standard-deviation uncertainty in Table 3. Therefore, in the following section, we used this value rather than the largest off-diagonal component of the sensitivity matrix to assign type B uncertainties to the off-diagonal components of the sensitivity matrix.

The magnitudes of the remaining errors in the sensitivity matrix and intrinsic properties were less than the corresponding standard-deviation uncertainties in Tables 3 and 4, and the latter values guided the assignment of uncertainties of these components.

## 5 Assignment of Uncertainties

The standard uncertainties assigned to the components of the sensitivity matrix and the intrinsic properties were as follows:

· *u*(*s_jk_*) = 2 × 10^-5^|*s_jk_*| for *j* = *k*,

· *u*(*s_jk_*) = 2 × 10^-4^|*s_jk_*| for *j*≠*k*,

· *u*(**s***_j_*) = 1 × 10^-5^|**s** *_j_*|,

· *u*(***O****_j_*) = 5 × 10^-5^|***O****_j_*|,

· *u*(***ϕ****_j_*) = 5 × 10^-3^|***ϕ****_j_*|,

where *j* ∈ {*u*, *v*, *w*}, and *k*∈{*x*,*y*,*z*}.

The values for **s***_j_*, ***O****_j_*, and ***ϕ****_j_* and the value for *s_jk_* are greater than or approximately equal to the greatest value in each category in Tables 3 and 4. These bounds also apply to the simulations in Appendix A. The value of *s_jk_* for *j* ≠ *k* comes from the simulations in simulations set ABC2. The listed values include uncertainties for ${\hat{Z}}_{X},{\hat{Z}}_{y},\text{and }{\hat{X}}_{z}$ and $f_{J}^{i}(1),...,f_{J}^{i}(N)$. (Recall that *j* indexes different accelerometers within the AUT and *J* indexes different axes of the ACS.)

## 6 Conclusion

We described a simulation methodology designed to estimate errors in the sensitivity matrix of triaxial accelerometers mounted on a two-axis ACS and measured and analyzed with the ABC sine-fitting protocol. We illustrated its use to isolate the effect of some individual ACS errors on the sensitivity matrix. We also used it to estimate type B standard uncertainties to describe the cumulative effect of all ACS errors on the sensitivity matrix associated with the use of the ABC protocol except nutation and stress-induced distortion of the instrument during operation. The results demonstrated that the simulated ACS imperfections cause errors in the sensitivity matrix calculated by the protocol whereas simulated AUT intrinsic-properties errors do not. Characterization experiments beyond those included in the ABC sine fitting protocol will be needed to characterize accelerometer non-linearity, temperature sensitivity, etc.

We expect the type B estimates given here to be conservative for three reasons. First, we used the manufacturer's 0.008° "accuracy" specification rather than its 0.001° "resolution" specification in the simulations. Second, we used this value as the standard deviation of 75 different independent distributions of ACS-associated errors that we identified. Third, simple experiments referred to in Appendix A showed that the only two errors that we have been able to measure so far fall well within one standard deviation of the mean of distributions we used.

Since no comparisons with independent measurements have been carried out so far, further caution is justified. Nevertheless, the resulting uncertainty estimate should be more than adequate for measurements requiring uncertainties no smaller than a few tenths of a percent. Furthermore, as the need for lower, more robust uncertainties grows with the maturation of MEMS triaxial-accelerometer technology, we expect to be able to replace these type B uncertainty estimates with lower-uncertainty Type-A estimates as outlined below.

The first step is to develop a method to accurately measure *f_J_*(*n*) for *J*∈{*X*, *Z*} and *n* = 1,..., *N*, as well as ${\hat{Z}}_{X},{\hat{Z}}_{y},\text{and}{\hat{X}}_{z}$ with Type-A uncertainties. If and when this task is complete, the next step is to use the entire simulation script as the "function" in a non-linear fitting algorithm in a massive set of Monte Carlo experiments like those in Simulations Set ABC1 and ABC2 to estimate the mean and standard error of the sensitivity matrix and zero-excitation offset triple ***O***.

For each Monte Carlo experiment

· The Monte Carlo experiment would start with the initial values for the elements of the sensitivity matrix and ***O*** that had been determined with the conventional ABC protocol.
· The Monte Carlo experiment would draw values of${\hat{Z}}_{X},{\hat{Z}}_{y},\text{and}{\hat{X}}_{z}$, and of each$f_{J}^{i}(n)$ from normal random distributions with the corresponding means set to the measured means, and the corresponding standard deviations set to the standard errors of the means obtained in the measurements of the different ACS errors.
· The fitting algorithm would minimize the differences between the measured and simulated response data for each AUT accelerometer in a least-squared sense, with ${\hat{Z}}_{X},{\hat{Z}}_{y},\text{and}{\hat{X}}_{z}$, and each $f_{J}^{i}(n)$set to the corresponding measured value. This step, which would be repeated a statistically significant number of times, would require running a new simulation of each rotation experiment each time the fitting algorithm tests a new combination of the nine elements of the sensitivity matrix and the three elements of ***O*** in an attempt to decrease the difference between the simulated and measured response data.
· The Monte Carlo experiment would generate new values for the elements of the sensitivity matrix and ***O*** corresponding to the random values of ${\hat{Z}}_{X},{\hat{Z}}_{y},\text{and}{\hat{X}}_{z}$, and each$f_{J}^{i}(n)$ being used at this step.

When all Monte Carlo experiments were complete, the means and standard errors of the means of the means of the sensitivity matrix and ***O*** obtained from each Monte Carlo experiment would be calculated and reported as the values and type A standard uncertainties derived from the determination. 

## 7 Appendix A: Properties of ${\hat{Z}}_{X},{\hat{Z}}_{y},\text{and}{\hat{X}}_{z}$

**Simulation Set 3:** This set tested the effect of imperfect orthogonality of the *$\hat{Z}$* and $\hat{X}$ axes in the ACS following construction of the ACS when measuring an ideal AUT. The procedure used with Simulation Set 1 was also used here with the single change that *${\hat{X}}_{z}$* = sin(0.008°) instead of zero. The simulated errors are shown in Table 5.

**Table 5:** Simulated errors in sensitivity matrix and associated intrinsic property triples of an ideal AUT measured with an otherwise ideal ACS for which *${\hat{X}}_{z}$* = 1.3963 × 10^-4^ = sin(0.008°).

Errors in Sensitivity Matrix and Associated Intrinsic Properties Triples					
Matrix	Vector	*x* Component	*y* Component	*z* Component	Unit
	${\overset{➝}{s}}_{u}$	-9.748e-09	-6.981e-05	-6.981e-05	R/g_233_
***s***	${\overset{➝}{s}}_{v}$	+6.981e-05	-9.748e-09	-6.981e-05	R/g_233_
	${\overset{➝}{s}}_{w}$	-9.252e-18	+9.252e-18	-1.950e-08	R/g_233_
	Triple	*u* Component	*v* Component	*w* Component	Unit
	**s**	-4.874e-09	-4.874e-09	-1.950e-08	R/g_233_
	***O***	+4.654e-05	+4.654e-05	+1.950e-08	R
	***ϕ***	-2.793e-07	+4.000e-03	+4.000e-03	Degrees

The range of the ratios of the absolute values of the errors in the default sensitivity matrix in Table 5 is enormous: There is a gap of almost 13 orders of magnitude between the largest and smallest errors and a gap of over two orders of magnitude between the largest error and the second largest error. Moreover, four of nine errors have the same value, which is that of the largest error, and is equal to 1.5 times ***O****_u_* and ***O****_v_*. Finally, each of the components of the sensitivity-matrix error is proportional to *${\hat{X}}_{z}$* over a range of simulations from arcsin*$\left({\hat{X}}_{z}\right)$* = 0.0008° to 0.08^-^ to well within 1 part in 10^-3^. The errors in the intrinsic sensitivity triples are all negligible compared to the dominant error in the sensitivity matrix for the three cases studied.

**Simulation Set 4:** This set tested the effect of imperfect orthogonality of the $\hat{Z}$ and $\hat{X}$ axes in the ACS following construction of the ACS when measuring the AUT for which the default sensitivity matrices were randomly generated as described in connection with Simulation Set 2. The procedure used with Simulation Set 2 was also used here with the single change that *${\hat{X}}_{z}$*= sin(0.008°) instead of zero. The simulated errors are shown in Table 6.

**Table 6:** Simulated errors in sensitivity matrix and associated intrinsic property triples of the randomly generated AUT defined in Table 2 measured with an otherwise ideal ACS for which *${\hat{X}}_{z}$* = 1.3963 × 10^-4^ = sin(0.008°).

Errors in Sensitivity Matrix and Associated Intrinsic Properties Triples					
Matrix	Vector	*x* Component	*y* Component	*z* Component	Unit
	${\overset{➝}{s}}_{u}$	-3.299e-06	-7.572e-05	-7.243e-05	R/g_233_
***s***	${\overset{➝}{s}}_{v}$	+6.424e-05	-2.579e-05	-9.003e-05	R/g_233_
	${\overset{➝}{s}}_{w}$	+1.842e-05	+8.189e-06	-1.024e-05	R/g_233_
	Triple	*u* Component	*v* Component	*w* Component	unit
	**s**	-2.487e-06	-2.494e-05	-9.661e-06	R/g_233_
	***O***	+4.829e-05	+6.002e-05	+6.833e-06	R
	***ϕ***	+7.748e-04	+4.484e-03	+3.740e-03	Degrees

Replacement of the ideal AUT by the randomly generated AUT of Table 2 changed all of the sensitivity-matrix errors and eliminated all of the regularities evident in the sensitivity matrix in Table 5. On the other hand, each component of the sensitivity-matrix error was still approximately proportional to *${\hat{X}}_{z}$* over a range of simulations from arcsin(*${\hat{X}}_{z}$*) = 0.0008° to 0.08°, but only to 1 part in 10^2^. This time, one of the errors in the intrinsic sensitivity triples was approximately 30% of that of the largest component of the sensitivity triple.

**Simulation Set 5:** This set tested the effect on the sensitivity matrix of an ideal AUT caused by imperfect alignment of the *Z* axis of the ACS with the *z* axis of the LGCS following installation of the ACS in our laboratory. The procedure used with Simulation Set 1 was also used here with the single change that ${\hat{Z}}_{X}={\hat{Z}}_{y}$= 1.3963 × 10^-4^ = sin(0.008°) instead of zero. The simulated errors are shown in Table 7.

**Table 7:** Simulated errors in sensitivity matrix and associated intrinsic property triples of an ideal AUT measured with an otherwise ideal ACS for which ${\hat{Z}}_{X}={\hat{Z}}_{y}$= 1.3963 × 10^-4^ = sin(0.008°).

Errors in Sensitivity Matrix and Associated Intrinsic Properties Triples					
Matrix	Vector	*x* Component	*y* Component	*z* Component	Unit
	${\overset{➝}{s}}_{u}$	+2.220e-16	+7.601e-18	+1.396e-04	R/g_233_
***s***	${\overset{➝}{s}}_{v}$	+1.950e-08	-1.110e-16	-1.485e-17	R/g_233_
	${\overset{➝}{s}}_{w}$	-2.094e-04	-6.981e-05	+8.882e-16	R/g_233_
	Triple	*u* Component	*v* Component	*w* Component	Unit
	**s**	-9.748e-09	-1.110e-16	-1.462e-08	R/g_233_
	***O***	-1.124e-17	+2.030e-17	-4.654e-05	R
	***ϕ***	+0.000e+00	+4.000e-03	+4.000e-03	Degrees

Most of the errors in the components of the default sensitivity matrix obtained from the ABC protocols were negligible relative to 10^-15^, but three were greater than or equal to the greatest for *${\hat{X}}_{z}$* = 1.3963 × 10^-4^ = sin(0.008°). Some regularities remained.

**Simulation Set 6:** This set tested the effect on the sensitivity matrix of randomly generated AUTs having imperfect alignment of the *Z* axis of the ACS with the *z* axis of the LGCS following installation of the ACS in our laboratory. The procedure used with Simulation Set 2 was used here with the single change that ${\hat{Z}}_{X}={\hat{Z}}_{y}$ = 1.3963 × 10^-4^ = sin(0.008°) instead of zero. The simulated errors are shown in Table 8.

**Table 8:** Simulated errors in sensitivity matrix and associated intrinsic property triples of the randomly generated AUT defined in Table 2 measured with an otherwise ideal ACS for which ${\hat{Z}}_{X}={\hat{Z}}_{y}$ = 1.3963 × 10^-4^ = sin(0.008°).

Errors in Sensitivity Matrix and Associated Intrinsic Properties Triples					
Matrix	Vector	*x* Component	*y* Component	*z* Component	Unit
	${\overset{➝}{s}}_{u}$	-1.081e-05	-7.183e-05	+8.521e-05	R/g_233_
***s***	${\overset{➝}{s}}_{v}$	-1.098e-06	-4.353e-05	-3.076e-05	R/g_233_
	${\overset{➝}{s}}_{w}$	-1.549e-04	-4.975e-05	-2.576e-05	R/g_233_
	Triple	*u* Component	*v* Component	*w* Component	unit
	**s**	+4.242e-05	+4.158e-05	-3.190e-05	R/g_233_
	***O***	-4.766e-06	-4.773e-05	-1.850e-05	R
	***ϕ***	+1.484e-03	+8.584e-03	+7.161e-03	Degrees

Replacement of the ideal AUT by the randomly generated AUT of Table 2 changed all of the sensitivity-matrix errors and the largest component of the sensitivity-matrix error. However, ${\overset{➝}{s}}_{w}$ was equal to 2***s****_u_*, and each component of the sensitivity-matrix error was proportional to ${\hat{Z}}_{X}(={\hat{Z}}_{y})$ over a range of simulations from ${\hat{Z}}_{X}$= sin(0.0008)° to sin(0.08)° to with ±1%. The latter result was expected based on the earlier results reported here.

**Consistency with experiments to determine** ***Z_x_* and *Z_y_*:** We carried out preliminary rotation experiments around *Z* with Θ*_X_* = Θ*_Z_* = 0, which we will describe elsewhere. These provided an estimate of $\Delta_{Z}=\sqrt{{\hat{Z}}_{X}^{2}+{\hat{Z}}_{y}^{2}}$ that gives Δ*_Z_* = 5 × 10^-5^ < 1.3963 × 10^-4^ = sin(0.008)^-^ with a standard-error of 1 × 10^-5^. Since the dominant errors in the sensitivity matrix scaled with *Z_x_* and *Z_y_*, our previous simulations based on the manufacturer's accuracy statement overestimate the dominant errors in the sensitivity matrix due to these two ACS errors by a factor of approximately 3.

We have not yet determined how to accurately measure or otherwise account for rotation-step errors. Because a rotation experiment to determine ${\hat{X}}_{Z}$ depends upon knowledge of the rotation-step errors at integer multiples of 90°, we have not yet carried out this type of experiment either. Note that it is not possible to determine rotation step errors from repeated measurements that quantify a systematic component of the residuals of the ABC fits to the response data because non-linearity of the accelerator sensitivity will also contribute systematic components to the residuals. Decomposing sensitivity-matrix errors into those caused by accelerometer non-linearity and those caused by repeatable rotation-step errors is beyond the scope of this report.

## 8 Appendix B

Addressing complexities associated with rotation around non-orthogonal axes resulted in notation changes between this paper and Ref. [2], which are summarized in the Table 9.

**Table 9:** Major difference in notation between notation in this work and the notation in [2].

Here	Ref. [2]	Quantity
*n*	*i*	Rotation-step index in rotation experiment
* $r_{iu}(\alpha (n))$ * * $r_{iv}(\alpha (n))$ * * $r_{iw}(\alpha (n))$ *	* $U^{P}(\alpha_{i})$ * * $V^{P}(\alpha_{i})$ * * $W^{P}(\alpha_{i})$ *	Response of U accelerometer at rotation step *n* ↔ *i* in rotation experiment↔ *i* ↔ *p* Response of V accelerometer at rotation step *n* ↔ *i* in rotation experiment *i* ↔ *p* Response of W accelerometer at rotation step *n* ↔ *i* in rotation experiment *i* ↔ *p*
*i* = 1 *i* = 2 *i* = 3	*p* = *x * *p* = *y* *p* = *z*	Index of first rotation experiment Index of second rotation experiment Index of third rotation experiment
